# Targeting pathogen sterols: Defence and counterdefence?

**DOI:** 10.1371/journal.ppat.1006297

**Published:** 2017-05-18

**Authors:** Kemal Kazan, Donald M. Gardiner

**Affiliations:** 1 Commonwealth Scientific and Industrial Research Organisation (CSIRO) Agriculture and Food, St. Lucia, Queensland, Australia; 2 Queensland Alliance for Agriculture & Food Innovation (QAAFI), the University of Queensland, St. Lucia, Queensland, Australia; THE SAINSBURY LABORATORY, UNITED KINGDOM

## Introduction

Sterols, a class of lipids found in the cellular membranes of all eukaryotes, have vital roles in regulating membrane fluidity and permeability. While animal cells contain cholesterol in their membranes, plant cells contain phytosterols such as campesterol, sitosterol, and stigmasterol. The sterol ergosterol, discovered more than a century ago from the ergot fungus *Claviceps purpurea* [1], is a common component of many plant and human pathogenic fungi and is required for fungal growth. Fungal plant pathogens—in particular, the smut (Ustilaginomycotina subphyla) and the powdery mildew (Pezizomycotina subphyla) fungi—contain ergosterol in their membranes, while the rust fungi (Pucciniomycotina subphyla) synthesise slightly different forms of sterols [2]. In contrast to most pathogenic fungi, the oomycete pathogens from the order Peronosporales, such as *Phytophthora* and *Pythium*, are sterol auxotrophs. Therefore, these organisms acquire sterols externally, most likely from host membranes during pathogenesis [3].

## Targeting ergosterol or its biosynthesis by fungicides provides effective but temporary protection against pathogens

**Fig 1:**
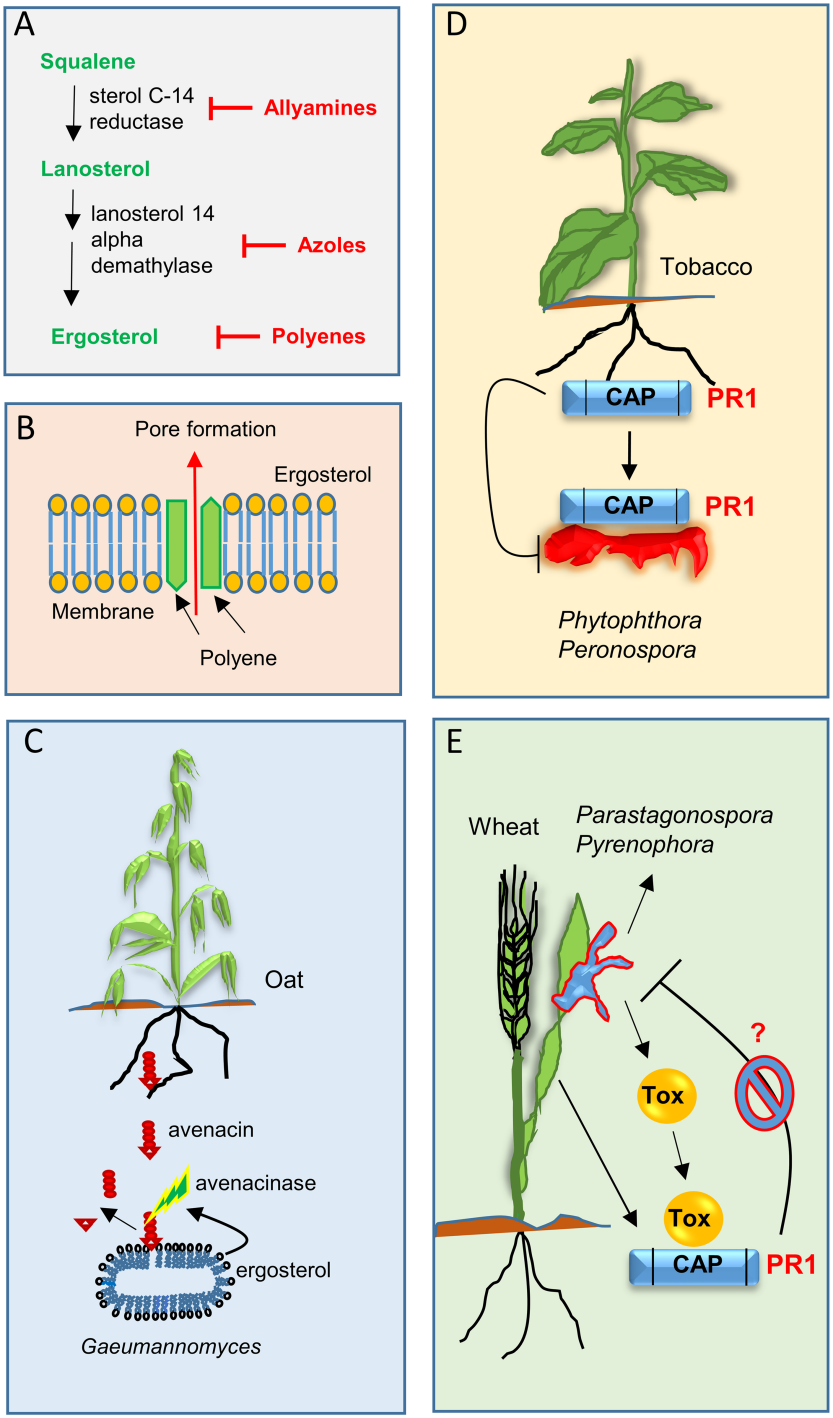
Different classes of fungicides (e.g., allyamines, azoles, and polyenes) inhibit pathogen growth by targeting the ergosterol biosynthesis pathway. Allyamines and azoles inhibit key enzymes in ergosterol biosynthesis required for membrane function (**A**), while polyenes disrupt membrane function, leading to pore formation by binding to ergosterol (**B**). Different classes of plant sterol binding proteins targeting pathogen cell membranes are important components of plant defence. In return, fungal pathogens have evolved to overcome such defences. Avenacin disrupts membrane integrity by forming complexes with sterols. The enzyme avenacinase, secreted by the fungal pathogen *Gaeumannomyces graminis* var. *tritici*, degrades avenacin, a saponin-type sterol binding protein secreted by oat roots (**C**). The pathogenesis-related protein 1 (PR1) from tobacco binds membrane sterols through its C-terminal cysteine rich secretory protein/antigen 5/PR1 (CAP) domain and provides pathogen protection by sequestering sterols from membranes of the oomycete pathogens *Phytophthora parasitica* and *Peronospora tabacina* **(D)**. A speculative model depicting how fungal wheat pathogens can potentially overcome PR1-mediated defences. Toxin (Tox) effectors (e.g., ToxA or SnTox3) secreted by *Parastagonospora nodorum* and *Pyrenophora tritici* var. *repentis* bind to the CAP domain of PR1, potentially (?) interfering with its sterol binding-mediated antifungal activity (**E**).

Realising the importance of sterols for fungal growth has led to the development of antifungal chemical compounds that target pathogens’ membrane sterols or sterol biosynthesis (Fig 1A and 1B). For instance, direct binding of polyene antibiotics, a class of antifungal compounds produced by *Streptomyces* bacteria, to membrane sterols causes pore formation and subsequent cell death in fungi [4] (Fig 1B). The antifungal compounds’ polyenes (e.g., aminopterin) are more specific to ergosterol than cholesterol and thus have been widely used to treat fungal infections in animals.

Azole group fungicides, another major classes of antifungal compounds commonly used to control animal (and a wide range of plant) pathogenic fungi, block sterol biosynthesis by inhibiting the cytochrome P450 monooxygenase (CYP51) lanesterol 14 alpha- demethylase involved in converting lanesterol to ergosterol [5] (Fig 1A). However, fungal pathogens often develop resistances to azole fungicides in a number of ways, including mutating or overexpressing the genes encoding CYP51 or various detoxification proteins (e.g., ATP-binding cassette [ABC] transporters) to reduce the intracellular accumulation of these toxic compounds [5].

Similarly, the inhibition of ergosterol biosynthesis through host-induced silencing of fungal lanesterol 14 alpha-demethylases provides significant protection against *Fusarium graminearum*, the causative agent of the Fusarium head blight disease in wheat [6]. The durability of this resistance is unknown, as mutations in RNA interference (RNAi)-targeted pathogen genes can render this type of protection ineffective. Therefore, multiple genes involved in ergosterol biosynthesis have been simultaneously targeted to increase both the effectiveness and the durability of RNAi-mediated protection against *F*. *graminearum* [6]. The intrinsic resistance observed in *F*. *graminearum* to amine fungicides (Fig 1A) also seems to result from the duplication of a pathogen gene encoding a sterol C-14 reductase enzyme involved in ergosterol biosynthesis [7].

## Innate plant defences target pathogen sterols as well

Given the importance of sterol/ergosterol for pathogen membranes, it is probably not surprising that plants have evolved capabilities to target this membrane component. For instance, saponins produced by certain plant species act to compromise plasma membrane integrity by interacting with 3β-hydroxyl sterols (Fig 1C). Saponins seem to be ineffective against oomycete pathogens due to the lack of 3β-hydroxyl sterols in their membranes [8]. Avenacin, a triterpenoid saponin produced by diploid oat (*Avena strigosa*), is required for increased tolerance against root infecting fungal pathogens such as the take-all pathogen *Gaeumannomyces graminis var*. *tritici* and *Fusarium* spp. [9]. Similarly, α-tomatine, a saponin compound produced by tomato (*Lycopersicum esculentum*), is required for basal defence against pathogenic fungi [10]. Tomatidine produced by α-tomatine hydrolysis interferes with ergosterol biosynthesis by inhibiting the enzyme sterol C24 methyl transferase [11,12]. Defence-inducing effects were also attributed to α-tomatine degradation products [11]. There is also evidence that certain plant antimicrobial peptides target fungal ergosterol [13] or disrupt the interaction between fungal membrane spingolipids and ergosterol [14]. More recently, the inhibition of CYP51 involved in ergosterol biosynthesis has been predicted to be the antifungal mode of action of coumarin, a natural compound produced by certain plant species [15]. Therefore, targeting fungal sterols seems to be an effective host defence strategy to counter pathogen attack. Saponins, however, seem to be ineffective against oomycete pathogens due to the lack of 3β-hydroxyl sterols in their membranes [8]. It should be noted that plants also contain 3β-hydroxyl groups in their membranes, but saponin-producing species seem to protect themselves from the toxic effects of these compounds by producing substituted sterols, which reduces the amount of sterols with a free alcohol group [8], or by storing them in vacuoles ready to be activated following tissue damage [16].

## Pathogenesis-related protein 1 (PR1)—A well-known host protein with newly discovered roles in sterol binding

The ability to produce saponins is limited to some dicot species and only oats in grasses and cereals [17]. This raises the question as to whether other plant defence strategies would also target pathogen sterols. Recent evidence points to PR1 family proteins, which are induced in response to pathogen attack, as a widely distributed player in this process (Fig 1D).

Genes encoding PR1-like proteins are typically induced by the defence-associated plant hormone salicylic acid (SA) and are markers for systemic acquired resistance (SAR), a plant immune response that provides broad spectrum protection against diverse pathogens. Interestingly, ergosterol is considered a microbe-associated molecular pattern (MAMP) molecule, triggering the expression of *PR* genes, including *PR1* [18]. The basic PR1 protein from tobacco increases the tolerance of tobacco (*Nicotiana tabacum*) to oomycete pathogens *Peronospora tabacina* and *Phytophthora parasitica* var. *nicotianae* when expressed transgenically [19]. However, the PR1 mechanism of action has remained enigmatic, despite the use of this gene as a marker in many studies since its discovery nearly half a century ago [20]. Interestingly, a recent study has shown a role for PR1 in sterol binding [21]. Two separate PR1 isoforms from tobacco, PR1a (an acidic, secreted PR1 protein) and P14c (a basic vacuolar PR1 protein), can bind and export cholesteryl acetate through the secretory pathway in the yeast *pry1pry2* double mutant, where cholesteryl acetate export is otherwise completely blocked [21]. Tobacco PR1 proteins bind sterols through their conserved CAP domain, which is required for their antifungal activity [21]. This capability is conserved in other C-terminal cysteine rich secretory protein/antigen 5/PR1 (CAP)-domain superfamily and pathogen-related yeast (PRY) proteins [22]. CAP-domain-containing secreted proteins are widely distributed in different taxonomic groups and have been implicated in both immunity- and virulence-related functions [23–27]. However, currently, very little is known about the molecular mode of action of such proteins.

Tobacco PR1 proteins seem to be particularly effective against sterol auxotroph oomycetes, most likely through sequestering sterols from their membranes [21]. Although the antimicrobial effects of tobacco PR proteins on fungal pathogens such as *Aspergillus niger* and *Botrytis cinerea* are weaker than they are on oomycetes, the efficacy of these proteins could be significantly increased when administrated together with sublethal doses of a chemical sterol biosynthesis inhibitor [21]. Therefore, antimicrobial effects of PR1 proteins seem to be dependent on the sterol biosynthetic capacity of different microbes. Nevertheless, extracellular or vacuolar locations of different PR1 proteins make them effective components of basal defence, directly countering pathogens either in the extracellular spaces or upon disruption of host cellular structures during infection. Given their ability to bind sterols, PR1 proteins can potentially affect plant membranes as well. However, plants most likely protect themselves from PR1 autotoxicity by synthesising these proteins in a pathogen-inducible manner and/or storing them in the vacuole until they are needed.

## Counterdefence strategies employed by pathogens neutralize host defences targeting sterols

Pathogens constantly develop new strategies to neutralise or overcome host defences. Therefore, it is not surprising that pathogenic fungi have evolved to counter host defences targeting their cellular membranes. The fungal enzymes avenacinase and tomatinase (produced by *G*. *graminis* var. *avenae*) and the tomato wilt pathogen *F*. *oxysporum* f. sp. *lycopersici* degrade avenacin and tomatine, respectively [17] (Fig 1C). This suggests that pathogens fight back against detrimental effects of saponins. Interestingly, a role for elicitins—MAMP molecules from oomycete pathogens (e.g., *P*. *infestans*)—in binding to host sterols is also known [28, 29]. However, the exact function of such modification on pathogen virulence is not entirely clear.

Therefore, an intriguing question would be if plant pathogenic fungi have also developed ways to overcome PR1-dependent defences targeting their sterols. Although no firm evidence for this currently exists, 2 recent papers showing interactions between unrelated toxin effectors from 2 separate wheat pathogens and the CAP domain of wheat PR1 proteins suggest that this might be a possibility (Fig 1E). The proteinaceous effector ToxA secreted by *P*. *tritici repentis*, the causative agent of the tan spot disease in wheat, interacts with the basic PR1 protein TaPR-1-5 [30]. Similarly, SnTox3, another toxin effector secreted by *Parastogonospora nodorum*, the causative agent of the wheat glume blotch disease, binds not only the basic PR1 protein TaPR-1-1 but also other acidic and basic members of the PR1 gene family that contains more than 60 members in the genome of hexaploid wheat [31]. However, it is currently unknown if binding by these effectors abolishes the antifungal mode of action of PR1 by interfering with the sterol/ergosterol binding activity of wheat PR1 proteins (Fig 1E).

ToxA and SnTox3 do not share any sequence conservation. Although both TaPR-1-5 and TaPR-1-1 belong to the group 1 basic PR1 proteins and share high sequence similarity, TaPR-1-5 and TaPR-1-1 CAP domain residues required for either ToxA or SnTox3 interactions seem to be different [30,31]. Also, the asparagine (N) 141 residue in the PR-1-5 protein, which interacts with the N102 residue in ToxA, is not a conserved part of the CAP domain. Rather, it is located in a unique sequence motif found only in wheat PR-1-5 and PR-1-4 but absent from other plant PR1 proteins [30]. Therefore, the suggestion that effector binding might interfere with sterol-binding activity of PR1 requires further investigation. Nevertheless, the 2 separate pathogen effectors having independently acquired an ability to interact with various members of the PR1 family, whose only known function in plant defence is sterol binding, is indeed intriguing.

## Do pathogens hijack PR1-mediated defences?

What makes PR1 even more interesting is that recent studies have identified a peptide, CAP-derived peptide 1 (CAPE1), that is derived from the C-terminal end of PR1 [32]. CAPE1 activates defence responses, suggesting that it may serve as a damage-associated molecular pattern (DAMP) molecule [32]. One intriguing possibility proposed by Breen et al. [31] is that effector (e.g., SnTox3) binding triggers the release of CAPE1 from PR1, which can then contribute to the development of tissue necrosis. Indeed, infection experiments conducted on wheat plants previously infiltrated with CAPE1 showed that this peptide can amplify the necrosis caused by SnTox3 in plants carrying the cognate susceptibility gene Snn3 [31]. Similarly, a mutant form of TaPR-1-5 (N141A) unable to interact with ToxA triggers reduced necrosis when infiltrated into wheat plants carrying the cognate susceptibility gene Tsn1 [30]. It should be emphasised, however, that these 2 fungal wheat pathogens hijack exaggerated host defence responses for their benefit. In other host pathogen interactions, such as the one between *Arabidopsis thaliana* and the bacterial pathogen *Pseudomonas syringae*, the activation of defence responses through the release of CAPE1 seems to play a beneficial role in restricting infection [32].

In conclusion, emerging evidence briefly reviewed in this paper indicates that plants produce secreted defence proteins that target sterols present in pathogen cell walls. In return, pathogens have developed innovative ways to render such defences ineffective. Future studies may reveal new clues about additional host proteins potentially involved in sterol binding and the strategies employed by pathogens to overcome such defences. Given the emerging link between azole fungicide use in agriculture and azole-resistant strains of both plant and human pathogens [33], this information will be useful in designing more effective and durable disease protection strategies in both plants and animals.
